# Determinants of comprehensive knowledge of HIV/AIDS among reproductive age (15–49 years) women in Ethiopia: further analysis of 2016 Ethiopian demographic and health survey

**DOI:** 10.1186/s12981-020-00305-z

**Published:** 2020-08-12

**Authors:** Chilot Desta Agegnehu, Bisrat Misganaw Geremew, Malede Mequanent Sisay, Kindie Fentahun Muchie, Zinash Teferu Engida, Temesgen Worku Gudayu, Daniel Sisay Weldetsadik, Alemneh Mekuriaw Liyew

**Affiliations:** 1 School of Nursing, College of Medicine and Health Sciences and Comprehensive Specialize Hospital, University of Gondar, Gondar, Ethiopia; 2 Department of Epidemiology and Biostatistics, College of Medicine and Health Sciences, University of Gondar, Gondar, Ethiopia; 3 Department of Epidemiology and Biostatistics, College of Medicine and Health Sciences, Bahir Dar University, Bahir Dar, Ethiopia; 4 Department of public health , School of Health Sciences, MaddaWalabu University, Bale Goba, Ethiopia; 5 Department of Clinical Midwifery, School of Midwifery, College of Medicine and Health Sciences, University of Gondar, Gondar, Ethiopia; 6 Epidemiology and Bio statistics unit, School of Public health, College of Medicine and Health Sciences, Dilla University, Dilla, Ethiopia

**Keywords:** Comprehensive knowledge, HIV/AIDS, Determinants, Ethiopia, Demographic and health survey

## Abstract

**Background:**

The key cause of HIV transmission is failure to provide adequate information about HIV/AIDS which is a substantial public health issue in low and middle-income countries. While global health coverage continues, there is still little understanding of HIV/AIDS among women of reproductive age (15–49 years) in Ethiopia. Therefore, the purpose of this study was to identify the determinants of comprehensive knowledge of HIV/AIDS among women of reproductive age in Ethiopia.

**Methods:**

A secondary data analysis was employed using the 2016 Ethiopian demographic and health survey data. Data were extracted about comprehensive knowledge of HIV/AIDS among women of reproductive age. We used multi-variable mixed-effect binary logistic regression to identify factors associated with comprehensive knowledge of HIV/AIDS among women of reproductive age. The adjusted odds ratio with 95% confidence interval was used to declare statistical significance.

**Results:**

We found that having primary (AOR = 1.75, 95% CI 1.56–1.97),secondary (AOR = 2.74, 95% CI 2.33–3.22), and higher (AOR = 4.07, 95% CI 3.32–4.99) educational statuses, being in highest wealth quintiles; richer (AOR = 1.20, 95% CI 1.01–1.43) and richest (AOR = 1.51, 95% CI 1.22–1.87), knowing the place for HIV test (AOR = 2.13, 95% CI 1.88–2.42), use of traditional contraceptive method (AOR = 1.93,95% CI 1.12–3.35), female household head (AOR = 1.18, 95% CI 1.07–1.31), watching television (AOR = 1.22, 95% CI 1.06–1.41) and own mobile phone (AOR = 1.18, 95% CI 1.05–1.33) were positively associated with comprehensive knowledge of HIV/AIDS among women of reproductive age in Ethiopia.

**Conclusion:**

Women with higher education and higher wealth quintiles, knowing the place of HIV test, watching television, a traditional contraceptive method use, having a mobile phone and being in female headed household were positively associated with comprehensive knowledge of HIV/AIDS among women of reproductive age in Ethiopia. Programs working on HIV/AIDS should target women based on the identified factors so as to scale up their comprehensive knowledge towards HIV/AIDS. In this context, the media should actively contribute to raising awareness of HIV/AIDS.

## Background

The acquired immunodeficiency syndrome (AIDS) due to human immunodeficiency virus (HIV) is still a global pandemic, particularly in low-income countries. Overall, 37.9 million people, including 36.2 million adults and 1.7 million children (< 15 years) were living with HIV worldwide at the end of 2018. Around 1.7 million have become newly infected and 770,000 people worldwide have died from AIDS related diseases. In general, adult women with HIV are more prevalent [1].

Lack of knowledge about HIV/AIDS is one of the main reasons for the increased number of new HIV infections and burdens for females of reproductive age (15–49 years), which is a major public health problem [2–6]. Globally, less than 30% of reproductive age women have comprehensive knowledge of HIV/AIDS [7]. Consequently, about 90% of the sub-Saharan African (SSA) countries were severely affected by HIV/AIDS, especially reproductive age women suffered from the HIV epidemic [8, 9]. Moreover, SSA countries contributed 70% of the new HIV infections where 74% of AIDS related deaths occurred [10].

There are significant challenges that remain to reach women of reproductive age living with HIV/AIDS for treatment and care services in SSA, accounting 59% of the total number of people living with HIV, in 2013 [10].Unlike other regions, the primary route of transmission in SSA is unsafe heterosexual intercourse that leads a young woman newly infected with HIV every minute. Studies revealed that young women were three to six times more likely to have HIV compared with males of the same age [11, 12].It is known that the level of HIV/AIDS related knowledge varies according to the socioeconomic status of the country [13].

According to the United Nations General Assembly Session (UNGAS) target set in 2001, 95% of young adults got to have correct and comprehensive knowledge of HIV/AIDS [14]. However, this is far below the target besides the higher prevalence of HIV/AIDS among females. Different studies conducted in Africa demonstrated that a low comprehensive knowledge of HIV/AIDS and less than half of reproductive age women demonstrated a comprehensive knowledge of HIV/AIDS. For example, in Mozambique, only 31.8% of women aged 15–49 years [15] and in northern Uganda, 23% of women of reproductive age [16] had a comprehensive knowledge of HIV/AIDS. Another study was done in three East African countries: Burundi, Kenya, and Ethiopia among women living with HIV/AIDS aged (15–49) also showed a comprehensive knowledge of HIV/AIDS was less than half in Burundi (48.9%) and Kenya (46.3%), while in Ethiopia it was below one-fourth(19.3%) [17]. According to the target, this indicates that it is a very low comprehensive knowledge of HIV/AIDS among women of reproductive age in Ethiopia.

Some studies also showed that lower educational level [18], poor wealth index [17, 19], and rural residence [20] and do not use contraceptives [21] were factors that decline comprehensive knowledge of HIV/AIDS among women of reproductive age. Having accurate HIV/AIDS knowledge about transmission and prevention is very important for avoiding HIV infection and ending the stigma and discrimination of infected and affected persons. However, studies have shown women of reproductive age lack accurate and complete information on how to avoid exposure to HIV.

Therefore, this study aimed to identify the potential factors associated with comprehensive knowledge of HIV/AIDS among women of reproductive age in Ethiopia based on the 2016 Ethiopian Demographic and Health Survey (EDHS) data.

## Methods

### Study design, setting, and period

Secondary data analysis was done from a community-based national cross-sectional study. The study was conducted in Ethiopia, which is situated in the Horn of Africa. It has 9 regional states (Afar, Amhara, Benishangul-Gumuz, Gambela, Harari, Oromia, Somali, Southern Nations, Nationalities, and People’s Region (SNNP) and Tigray) and two administrative cities (Addis Ababa and Dire-Dawa).

**Fig. 1:**
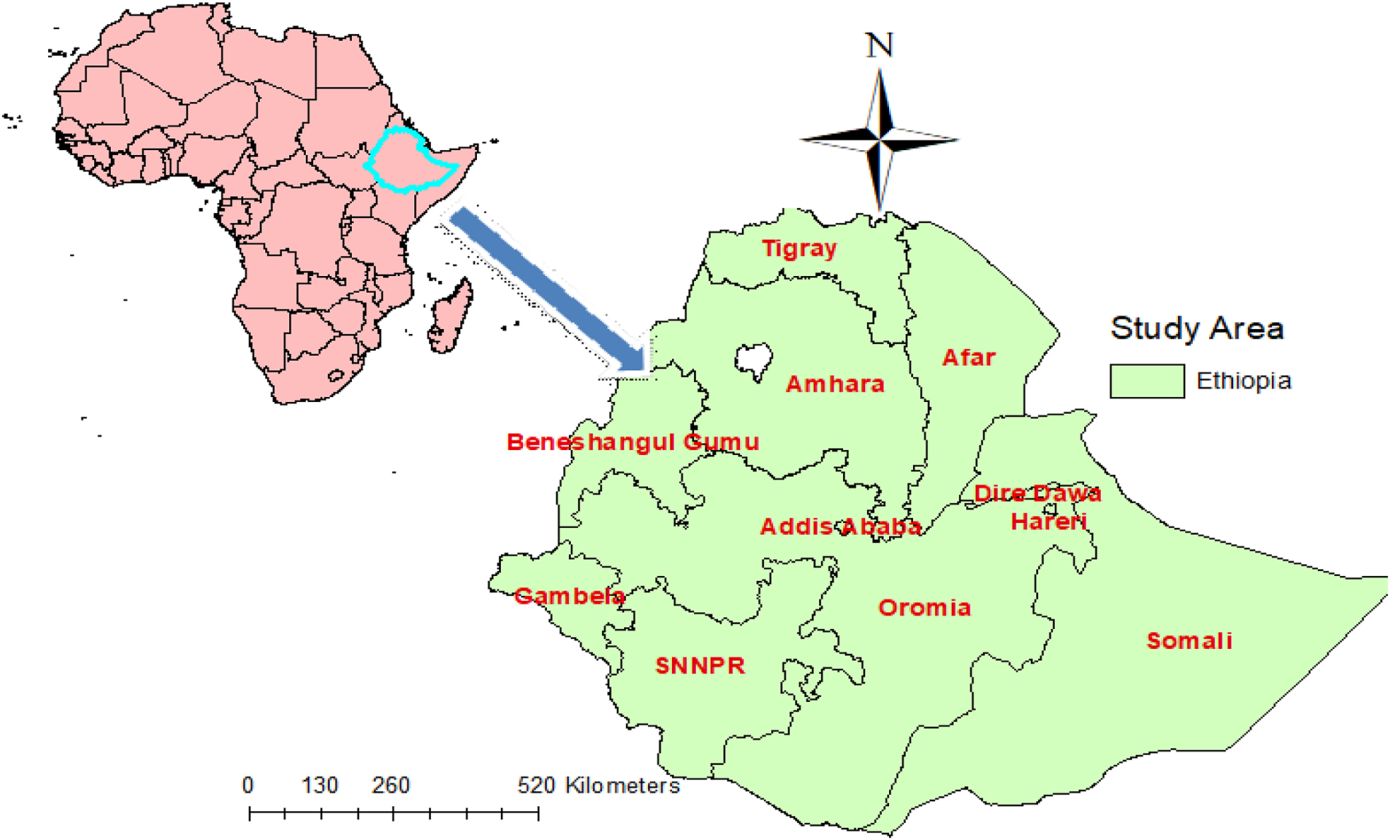
Map of study area Source: Shape file: central statistical Agency, 2016

In 2016, the population of Ethiopia was estimated as 102 million, of which 43.47% of the population is aged less than 14 years with a birth rate of 36.5 births per 1000 population and fertility rate of 4.46. Ethiopia is the 13th in the world and 2nd in Africa most populous country. The country has three tiers of health systems; primary health care units (primary hospitals, health centers, health posts, primary clinics, and medium clinics); secondary health care (general hospitals, specialty clinics, and specialty centers) and tertiary health care (specialized, teaching hospitals). In response to population differences, the number of hospitals varies from region to region. Oromia, which is the most densely populated region, has 30 hospitals. The other two regions with 19 and 20 respectively are Amhara and SNNPR. Although there were 16 hospitals in Tigray, there is only one hospital in Gambela, while there are two in Benishangul-Gumuz [22] (Fig. 1).

### Data source

The data for this study were taken from the 2016 EDHS. It is the fourth comprehensive and nationally representative survey conducted as part of the worldwide demographic and health surveys (DHS) project. The EDHS 2016 data were downloaded from the DHS website (http://dhsprogram.com) after getting authorization. The 2016 EDHS samples were selected using a stratified two-stage cluster sampling design. In the first stage, about 645 enumeration areas (EAs) (202 in urban areas and 443 in rural areas) were selected with probability proportional to the EAs size and with independent selection in each sampling stratum.

The study’s source population was all reproductive women aged 15–49 years and the study population was all women between 15 and 49 years in the selected EAs. A total of 14,369 women had been asked about HIV/AIDS knowledge. Potential predictors such as education, wealth index, place of residence (urban versus rural), the type of contraceptive, sex of household head and other variables were extracted from the dataset based on the availability of data and previous literature. More methodological details can be accessed from EDHS 2016 report [23].

### Study variables

The outcome variable is comprehensive knowledge of HIV/AIDS which is defined as correct knowledge of two mechanisms to prevent HIV and rejection of three misconceptions about HIV. To assess the comprehensive HIV and AIDS knowledge of reproductive age women, every woman was asked whether or not she correctly answered the following questions: (1) can using condoms prevent HIV transmission? (2) can HIV be prevented by limiting sex to one faithful uninfected partner? (3) can a person get HIV from mosquito bites? (4) can a person get HIV by sharing food with someone infected? and (5) can a healthy-looking person have HIV? [24]. Accordingly, comprehensive knowledge of HIV/AIDS coded as non-knowledge women = 0 and knowledge women = 1.

The independent variables of the study were drawn from socio-demographic characteristics (age, education status, residence, region, religion, household head sex), health care and system-related (method of contraception, know the place of the test for HIV) and socioeconomic factors (media exposure such as TV, reading newspapers, wealth index, own mobile phone).

### Data management and methods of analysis

After the extraction of data, STATA software Version 14 (StataCorp LP, College Station, Texas 77845 USA) was used to clean up, rename, recode, and further analysis. Sampling weights were used to adjust the probability of selection and non-response differences. First, we examined socio-demographic characteristics of the sample using descriptive statistics. Data were correlated (within-cluster correlation 0.26), as EDHS has a hierarchical and clustered structure. This requires the use of advanced model that considers variability due to clustering nature. A mixed effect binary logistic regression modeling under the broad family of generalized mixed modeling was therefore adapted. In the bivariable analysis, variables with p-values ≤ 0.2 were selected to adjust in the final model. Finally, the p-values less than 0.05 were used to classify variables with statistical significance of comprehensive knowledge of HIV/AIDS in the multivariable analysis.

## Results

### Socio-demographic characteristics of study participants

Almost one-fifth of participants (21.4%) were teenagers with three quarters (76.0%), were rural inhabitants (Table 1). Orthodox Christians accounted for around 44.8%. About 45.4% of the participant did not attend formal education and more than a quarter (28.2%) was in the rich wealth quantile. Of all 12,133 (83.0%) of the participants didn’t watch television. Whereas seventeen percent (17.2%) and twenty-nine percent (29.0%) of the participants had the experience of listening radio and owns mobile phones, respectively. Three quarters (74.5%) of the study participants knew the place of HIV test. Among the study participants, twenty-four percent (24.0%) were from female headed household.

**Table 1:** Socio-economic and demographic characteristics and comprehensive knowledge of HIV/AIDS among reproductive age (15–49 years) women in Ethiopia, EDHS 2016 (n = 14,598)

Variables	Knowledge of HIV/AIDS		Total frequency	Percent
	Knowledgeable	Not-knowledgeable		
Age (in years)				
15–19	871	2252	3123	21.4
20–24	759	1868	2627	18.0
25–29	696	2067	2763	18.9
30–34	520	1673	2193	15.0
35–39	410	1359	1769	12.1
40–44	231	964	1195	8.2
45–49	191	737	928	6.4
Residence				
Urban	1512	1925	3437	24.0
Rural	2166	8995	11,161	76.0
Religion				
Orthodox	2030	4504	6534	44.8
Catholic	7	106	113	0.8
Muslim	830	3447	4277	29.3
Protestant	787	2708	3495	23.9
Traditional	16	94	110	0.7
Others	8	61	69	0.5
Wealth index				
Poorest	314	1922	2236	15.3
Poorer	459	2060	2519	17.3
Middle	496	2265	2761	18.9
Richer	687	2280	2967	20.3
Richest	1721	2393	4114	28.2
Educational status				
No education	976	5656	6632	45.4
Primary	1378	3907	5285	36.2
Secondary	798	1007	1805	12.4
Higher	526	349	875	6.0
Region				
Tigray	347	765	1112	7.6
Afar	18	99	117	0.8
Amhara	997	2587	3584	24.6
Oromia	1149	3938	5087	34.8
Somali	19	294	313	2.1
Benishangul	26	120	146	1.0
SNNP	611	2542	3153	21.6
Gambela	11	29	40	0.3
Harari	9	28	37	0.2
Addis Ababa	470	455	925	6.3
Watching television				
No watch at all	2516	9617	12,133	83.0
Watch once a week	1162	1303	2465	17.0
Listening radio				
No, listen at all	2754	9343	12,097	82.8
Listen to the radio once a week	924	1577	2501	17.2
Owns mobile phone				
No	1936	8467	10,403	71.0
Yes	1742	2453	4195	29.0
Know the place of HIV test				
No	420	3 303	3723	25.5
Yes	3258	7617	10,875	74.5
Sex of household head				
Male	2548	8545	11,093	76.0
Female	1130	2375	3505	24.0

### Comprehensive knowledge of HIV/AIDS

**Fig. 2:**
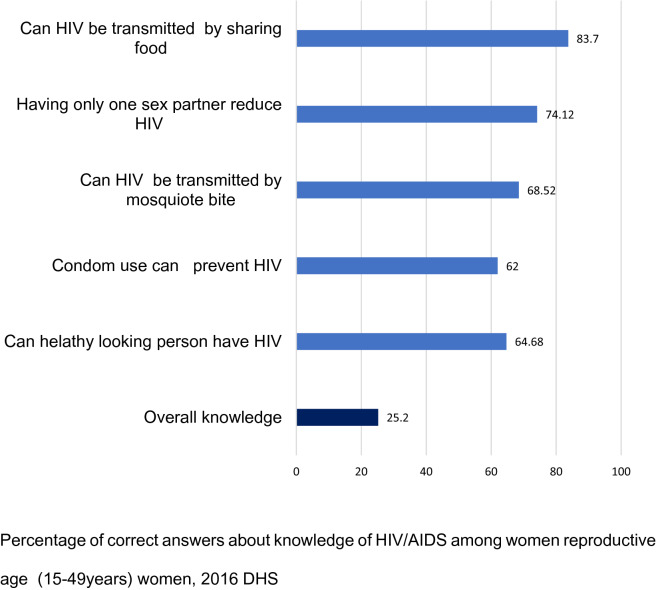
Percentage of correct answers about knowledge of HIV/AIDS among reproductive age (15–49 years) women, 2016 DHS

Approximately three-fourths (74.1%) and 68.5% indicated the risk of HIV and transmission could not be decreased by a single partner and a mosquito bite, respectively (Fig. 2). More than four-fifths (83.7%) of women (15–49) were aware of sharing foods with people who have HIV will not transmit HIV and 62.0% of women knew that condoms could help prevent sexual transmission of HIV. Nearly two-thirds of respondents (64.7%) had correctly answered the question of whether a healthy looking person could have HIV. Overall, only one quarter (25.2%) of Ethiopian women had a comprehensive knowledge of HIV/AIDS (95% CI 24.48–25.9).

### Determinants of comprehensive knowledge of HIV/AIDS among Ethiopian women

In the null model, variance component analysis was performed to decompose the total variance of comprehensive HIV/AIDS knowledge. The cluster-level variance which indicates the total variance of comprehensive HIV/AIDS knowledge that can be attributed to the context of the community (cluster) in which the women were living was estimated. The applicability of multi-level mixed-effects logistic regression model in the analysis was justified by the significance of the community-level variance [community variance = 1.16; standard error (SE) = 0.097; P-value = 0.001], indicating the existence of significant differences between communities (clusters) regarding the comprehensive HIV/AIDS knowledge. This was supported by the intra-cluster correlation coefficient (ICC) value (ICC = 0.26) which revealed that 26% of the total variance of comprehensive HIV/AIDS knowledge in Ethiopia can be attributed to the context of the communities where the mothers were dwelling. Since it was above the cut of point [25] the nuisance of clustering was considered to produce reliable estimates.

In bi-variable mixed-effect logistic regression; mother’s age, education level, high wealth index, knowing HIV test place, residence, contraceptive methods used, sex of household head, watching television, listening radio, reading newspapers, and own mobile telephone have been relevant at P-value < 0.2 and tailored to multi-variable analyses. However, we found that higher mothers educational level, knowing the site of HIV testing, sex of household head, listening radio, high-wealth indices, traditional methods of contraceptive use, possession of cell phones, and watching TV were statistically significant in relation with the comprehensive knowledge of the HIV/AIDS among women of reproductive age in Ethiopia.

The chances of having good comprehensive knowledge among women with primary education were 1.75 (AOR = 1.75, 95% CI 1.56–1.97) times higher than no education (Table 2). Similarly, being in secondary education level was more than twice as likely to have good knowledge (AOR = 2.74, 95% CI 2.33–3.22) and fourth times for higher education (AOR = 4.07, 95% CI 3.32–4.99) as compared to no educated women’s. We also found that richer women had 1.2 times higher chance of having good comprehensive knowledge as compared to the poorest (AOR = 1.20, 95% CI 1.01–1.43). And richest women had 1.5 times the chance to have good knowledge (AOR = 1.51, 95% CI 1.22–1.78) as compared to the poorest.

**Table 2:** Determinants of Comprehensive Knowledge of HIV/AIDS among reproductive age (15–49) women in Ethiopia

Variables	COR (95% CI)	AOR (95% CI)	P-value
Age (in years)			
15–19	1	1	
20–24	1.10 (0.97–1.25)	0.82 (0.93–1.07)	0.35
25–29	0.88 (0.77–0.99)	0.91 (0.79–1.04)	0.18
30–34	0.87 (0.76–1.00)	1.13 (0.96–1.32)	0.11
35–39	0.81 (0.70–0.94)	1.10 (0.94–1.29)	0.23
40–44	0.64 (0.54–0.76)	0.88 (0.72–1.06)	0.19
45–49	0.68 (0.56–0.83)	0.10 (0.83–1.26)	0.81
Educational level			
No education	1	1	
Primary	1.91 (1.73–2.11)	1.75 (1.56–1.97)**	< 0.001
Secondary	3.74 (3.27–4.27)	2.74 (2.33–3.22)**	< 0.001
Higher	6.41 (5.38–7.65)	4.07 (3.32–4.99)**	< 0.001
Wealth index			
Poorest	1	1	
Poorer	1.29(1.09–1.53)	1.14(0.96–1.36)	0.11
Middle	1.26(1.06–1.50)	1.02(0.85–1.21)	0.80
Richer	1.73(1.43–2.05)	1.20(1.01–1.43)**	0.04
Richest	3.55(2.98–4.23)	1.51(1.22–1.87)**	< 0.001
Know place of HIV test			
No	1	1	
Yes	2.55 (2.26–2.89)	2.13 (1.88–2.42)**	< 0.001
Residency			
Urban	3.70 (3.13–4.38)	1.14 (0.91–1.42)	0.23
Rural	1	1	
Method of contraceptive use			
Modern method	1.00 (0.91–1.10)	1.05 (0.95–1.16)	0.32
Traditional method	2.59 (1.53–4.37)	1.93 (1.12–3.35)**	0.018
Non users	1	1	
Sex of household head			
Male	1	1.	
Female	1.26 (1.15–1.39)	1.18 (1.07–1.31)**	< 0.001
Watching television			
No watch at all	1	1	
Watch once a week	2.35 (2.07–2.67)	1.22 (1.06–1.41)**	0.006
Listening radio			
No listen at all	1	1	
Listen radio once a week	1.57 (1.41–1.75)	1.08 (0.96–1.21)	0.18
Reading news paper			
No read at all	1	1	
Read once a week	1.84 (1.53–2.02)	1.09 (0.90–1.32)	0.35
Owns mobile phone			
No	1	1	
Yes	2.33 (2.11–2.57)	1.18 (1.05–1.33)**	0.04
Random component			
Community variance (SE)	–	1.16 (0.097)	
Intra-cluster correlation coefficient (ICC)		0.26 (0.23,0.29)	

** *p*-value < 0.05

Moreover, knowing the place of HIV test (AOR = 2.13, 95% CI 1.88–2.42), use of traditional contraceptive method (AOR = 1.93, 95% CI 1.12–3.35), being from female headed household (AOR = 1.18, 95% CI 1.07–1.31), Watching television (AOR = 1.22, 95% CI 1.06–1.41) and having owned mobile phone (AOR = 1.18, 95% CI 1.05–1.33) were remained positively associated with comprehensive knowledge about HIV/AIDS.

## Discussion

Among women of reproductive age, we found that higher educational level, Knowing the site of HIV testing, sex of household head, listening radio, high-income indices, traditional methods of contraceptive use, possession of cell phones, and watching TV were positively associated with comprehensive knowledge of HIV/AIDS.

Women’s education is positively associated with comprehensive knowledge of HIV/AIDS in Ethiopia. This result is supported by different studies done in three east African countries [17], Bangladesh [18, 21], Tajikistan [26], and Vietnam [27]. This might be because educated individuals will have more access to information regarding HIV/AIDS than their counterparts. Similarly, women being in the highest wealth index had a good comprehensive knowledge of HIV/AIDS as compared to the poorest women. This result is in agreement with the study done in Nigeria and Republic of Congo [20], in Ghana [19], in three east African countries [17]. This is due to the reason that having good socioeconomic status improves media exposure or educational achievement which increases the likelihood of knowledge about HIV/AIDS [28].

Knowing the place of HIV test was also positively associated with the comprehensive knowledge of women about HIV/AIDS. This is consistent with previous other studies among women in Vietnamese [27]. This might be due to those who know the place of HIV tests are more exposed to information and then more aware of HIV/AIDS from the counseling site. Moreover, provision of public education during health care services as a key component of primary prevention of AIDS has to be appreciated.

Media exposure increases the chance of comprehensive HIV/AIDS knowledge. The likelihood of having a comprehensive knowledge of HIV/AIDS among women who watched television once a week and who had a mobile phone was 1.22 and 1.18 times higher as compared to their counterparts respectively. This result is consistent with a study conducted in Bangladesh [18, 29]. This may be due to television is effective media to reach the general people which communicate important messages about this incurable disease in the form of music, news reports, dramas, movies, advertisements, etc. That can further highly influence public awareness about health and health-related issues including HIV/AIDS.

Regarding having mobile phone, those who owned mobile phone might get different information about HIV/AIDS from telecommunication staff. The results of this study pointed out that a health education program on AIDS for women greatly and significantly improved their knowledge of AIDS of transmission. One of the significant predictors for comprehensive knowledge of HIV/AIDS is a method of contraceptive use. This might be due to those women who use traditional contraceptive methods may be literate and then prone to information than non-users.

Regarding the sex of household head, being in a female headed household was positively associated with comprehensive knowledge of HIV/AIDS among women in Ethiopia. This study is in agreement with a study in Bangladesh [21]. Knowledge variation among genders could be due to the empowerment of women in education from time to time. As identified in the current study, many women were educated and the educational level of women was also positively associated with comprehensive knowledge of HIV/AIDS.

Ethiopia has amazingly performed in reducing burden of HIV/AIDS and to reverse this pandemic. Strong policy, leadership and political commitment to the creation and delivery of health programs that drive primary care are especially significant. Since 2003, Ethiopia has adopted the Health Extension Program (HEP) which is creative community-based health services [30]. Also, the extension, free of charge since 2003, of antiretroviral therapy (ART), may be instrumental in the HIV/AIDS incidence and mortality rate decrease [31]. Ethiopia now faces some challenges to achieve the 90 to 90–90–90 global HIV/AIDS targets by 2020, despite significant progress made in the past. Continuous mobilization of community groups, stigma reduction, and support services are therefore crucial to increased use of ART services and improved care retention that contributes to reducing early mortality from HIV/AIDS in Ethiopia.

This study is based on the EDHS 2016, which uses national sources of information and rigorous analyses. We used multi-level modeling for a better estimate of the impact of clustering between the related variables and the outcome variable. However, there are certain limitations to the study. The use of cross-sectional information can first lead to distortion. Secondly, a random error may occur in some independent variables, which have been subjectively defined such as the wealth index, even if the data collector is trained on a method and method of data collection.

## Conclusion

In general, women with the highest educational status and wealth index, watching television, having mobile phone, being in female headed household, using contraceptive method, and knowing place for HIV test appeared significantly associated with comprehensive knowledge of HIV/AIDS. The finding indicates that HIV prevention programs had contributed a lot to improve creating awareness and illustrate misconceptions regarding the mode of transmission of the disease. Media is very important tool to prevent any health-related problems by enhancing the knowledge of reproductive age women about HIV/AIDS. Therefore, Mass media and social workers have devised to transform ways to grow among people in the services providers and encourage service utilization. It is also better to promote prevention methods through mass media campaigns, and information, education, and communication programs to reproductive age women.

## Data Availability

All relevant data are available from the corresponding author upon a reasonable request

## Abbreviations

AOR: Adjusted odds ratio
AIDS: Acquired immunodeficiency syndrome
ART: Antiretroviral therapy
COR: Crude odds ratio
CI: Confidence interval
DHS: Demographic and Health Surveys
EAs: Enumeration sreas
EDHS: Ethiopian Demographic and Health Survey
HEP: Health Extension Program
HIV: Human immunodeficiency virus
ICC: Intra-cluster correlation coefficient
SNNP: Southern Nations, Nationalities, and People’s
SE: Standard error
SSA: Sub-Saharan African
TV: Television
UNGAS: United Nations General Assembly Session
